# Up-Regulated Expression of Thioredoxin-Interacting Protein (TXNIP) in Mesenchymal Stem Cells Associated with Severe Aplastic Anemia in Children

**DOI:** 10.3390/ijms252212298

**Published:** 2024-11-15

**Authors:** Ying-Hsuan Peng, Chang-Wei Li, Kang-Hsi Wu, Ju-Pi Li, Shun-Fa Yang, Yu-Hua Chao

**Affiliations:** 1 Department of Pediatrics, Chung Shan Medical University Hospital, Taichung 402, Taiwan; pengyh0823@gmail.com (Y.-H.P.); cshy1903@gmail.com (K.-H.W.); d888203@gmail.com (J.-P.L.); 2 Institute of Medicine, Chung Shan Medical University, Taichung 402, Taiwan; ysf@csmu.edu.tw; 3 Department of Research and Development, AllBio Life Incorporation, Taichung 402, Taiwan; vit@allbiolife.com; 4 School of Medicine, Chung Shan Medical University, Taichung 402, Taiwan; 5 Department of Medical Research, Chung Shan Medical University Hospital, Taichung 402, Taiwan; 6 Department of Clinical Pathology, Chung Shan Medical University Hospital, Taichung 402, Taiwan

**Keywords:** mesenchymal stem cells, severe aplastic anemia, thioredoxin-interacting protein

## Abstract

The pathogenic mechanisms of severe aplastic anemia (SAA) in children are not completely elucidated. The insufficiency of the bone marrow microenvironment, in which mesenchymal stem cells (MSCs) are an important element, can be a potential factor associated with hematopoietic impairment in SAA. In the present study, we compared bone marrow MSCs from five children with SAA and five controls. We found a higher intensity of senescence-associated β-galactosidase activity in SAA MSCs, indicating the increased senescence in these cells. Further RNA sequencing analysis identified a distinctive profile of transcriptomes in SAA MSCs. After conducting a survey of the differentially expressed genes, we found that the up-regulated expression of TXNIP may compromise the proliferative potential of MSCs and probably relate to the pathogenesis of SAA. These results were validated by qPCR. To explore the molecular mechanism involving aberrant TXNIP regulation in SAA MSCs, the expression levels of IGF-1 and IGFBP-1 were measured. A significant increase in IGFBP-1 expression was noted in SAA MSCs despite the wide range of IGF-1 expressions. Accordingly, we postulated a novel pathogenic mechanism of SAA: a compensated increase in the expression of IGF-1 in MSCs to down-regulate TXNIP expression in the face of SAA, which is offset by the up-regulated expression of IGFBP-1.

## 1. Introduction

Severe aplastic anemia (SAA), characterized by the failure of hematopoiesis, is a rare but potentially life-threatening disease in children with an annual incidence of 1–6 per million [1,2,3]. A specific cause cannot be detected in most children, and it is termed “idiopathic SAA”. Although immune-mediated hematopoietic stem cell destruction is considered an important factor [4], the pathogenic mechanisms of SAA have not been completely elucidated.

Mesenchymal stem cells (MSCs), first isolated by Friedenstein in 1966 [5], play an important role in establishing the specialized bone marrow microenvironment for hematopoiesis. The deficiency or dysfunction of MSCs may lead to bone marrow insufficiency and predispose to hematopoietic impairment, which is the main characteristic of SAA. As the clinical application of MSCs has evolved rapidly in recent years, the impact of MSC alterations on SAA has received more and more attention. In our previous study, we first demonstrated the poor proliferation and differentiation potential of bone marrow MSCs in children with SAA [6]. Many different approaches were taken to detect MSC defects in SAA, and alterations in their hematopoietic support, proliferation potential, differentiation capacity, immunomodulatory function, and gene expression were reported [7]. In line with this series of works, the present study aimed to find additional evidence for MSC insufficiency in the SAA bone marrow, focusing on aberrant gene expression and regulation.

## 2. Results

### 2.1. Patients and Controls

A total of five patients with SAA and five controls were enrolled in this study (Table 1). All participants were younger than 18 years old, and all bone marrow aspirates were collected during diagnosis without concomitant medication. The average ages of patients with SAA and the controls were 12.7 and 11.6 years, respectively. In patients with SAA, chromosome breakage analysis, flow cytometry, and cytogenetic studies were performed to screen for Fanconi anemia, paroxysmal nocturnal hemoglobinuria, and myelodysplastic syndrome. Secondary aplastic anemia following toxic exposure, infections, autoimmune disorders, and drugs were excluded. No specific causes were found for pancytopenia and bone marrow failure in all five patients with SAA.

### 2.2. Characterization of MSCs

**Figure 1:**
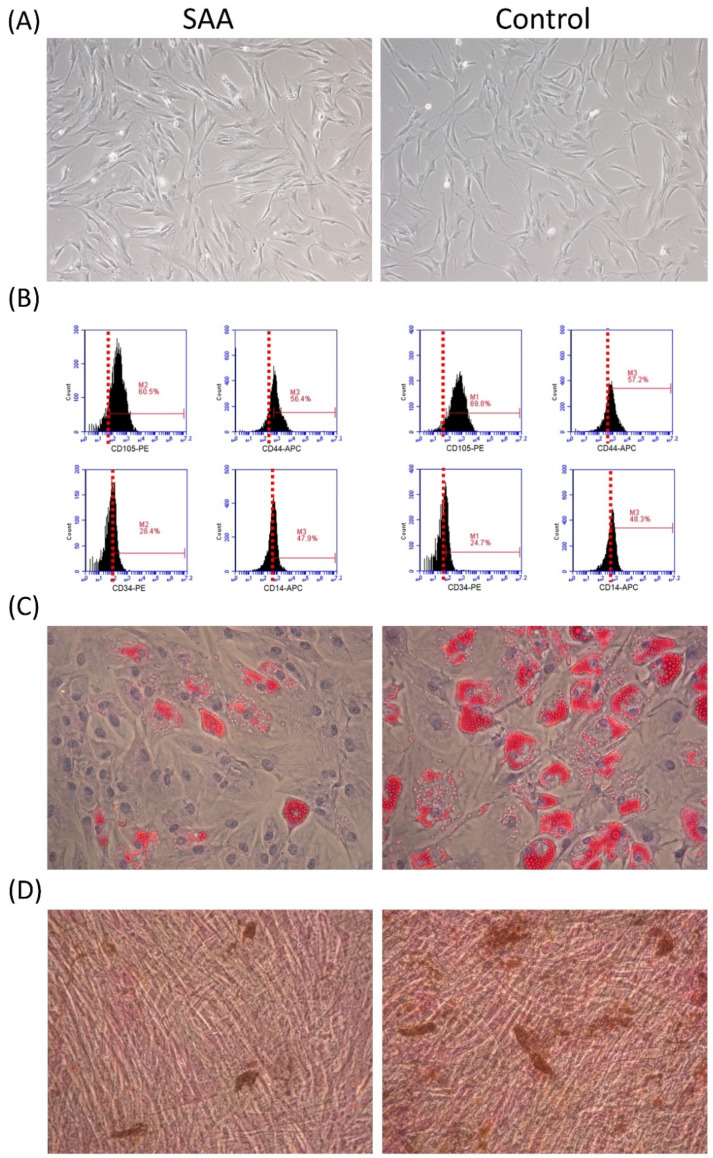
MSC identification. MSCs in both SAA and control groups were in accordance with the criteria of the International Society for Cellular Therapy. (**A**) Morphology (×200). (**B**) Immunophenotypic expression detected by flow cytometry. (**C**) Adipogenesis after 2-week adipogenic induction (oil red O staining, ×200). (**D**) Osteogenesis after 2-week osteogenic induction (von Kossa staining, ×200).

The criteria of the International Society for Cellular Therapy were used for MSC identification [8]. In vitro, MSCs from patients with SAA and controls share a similar spindle-shaped morphology and adhere to plates when maintained in culture conditions (Figure 1A). The MSCS in both groups expressed CD105 and CD44 and were negative for CD34 and CD14 (Figure 1B). There was no significant difference in the expression of any single surface marker between the two groups. Under induction conditions, MSCs in both groups could achieve adipogenic and osteogenic differentiation (Figure 1C,D). Consistent with our previous reports [6], adipogenesis and osteogenesis were less robust in SAA MSCs. These findings indicated that MSCs in both SAA and control groups were in accordance with the identification criteria.

### 2.3. Increased Cell Senescence in MSCs from Patients with SAA

**Figure 2:**
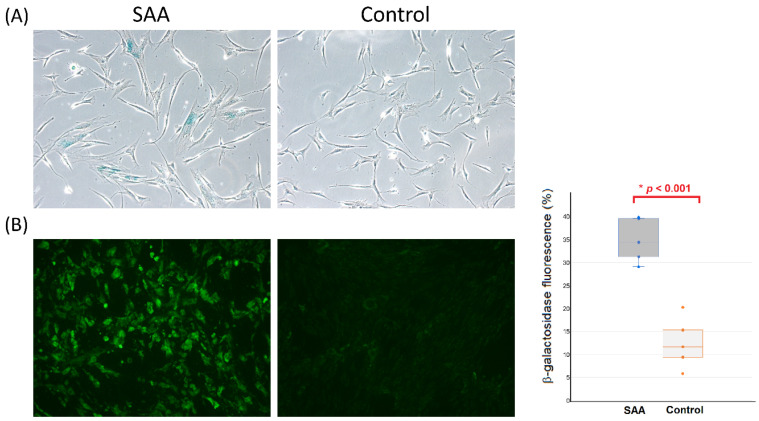
Comparison of senescence-associated β-galactosidase activity in MSCs between patients with SAA and controls. (**A**) Cytochemical staining (×200). (**B**) Fluorescence detection (×200) and quantification by flow cytometry. Each dot in the boxplot represents the intensity of β-galactosidase fluorescence for each sample. * Mann-Whitney U test.

β-galactosidase is a eukaryotic hydrolase localized in the lysosome that is active at an optimal pH in senescent cells but is absent in proliferating cells. Senescence-associated β-galactosidase activity is suggested as the gold standard for evaluating the intensity of senescence in cells [9,10]. After staining, a greater proportion of MSCs developed a blue color within cells in the SAA group, indicating the increased senescence in these cells (Figure 2A). The intensity of senescence-associated β-galactosidase activity by fluorescence detection was higher in MSCs from patients with SAA compared to MSCs from controls (*p* < 0.001; Figure 2B).

### 2.4. MSCs from Patients with SAA Showing a Distinctive Pattern of Transcriptomes

**Figure 3:**
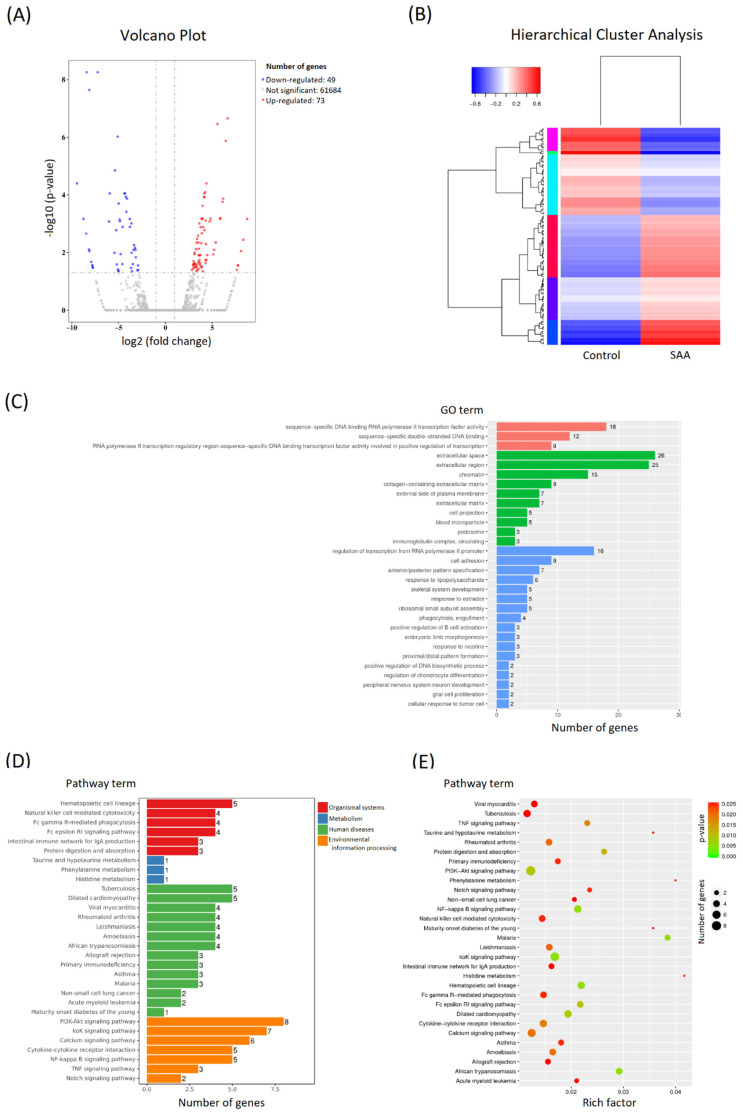
Comparison of gene expression profiling in MSCs between the patients with SAA and the control. (**A**) Differential expression volcano plot. Red dots represent genes that are significantly up-regulated, and blue dots represent genes that are significantly down-regulated. In total, 49 down-regulated genes and 73 up-regulated genes were significantly differentially expressed in the SAA sample. (**B**) Hierarchical cluster analysis of the differentially expressed genes. Up-regulated genes are in red, and down-regulated genes are in blue. (**C**) GO enrichment histogram. The number of differentially expressed genes in each GO term is illustrated with the specification of the relevant biological process (red), cellular component (green), and molecular function (blue). The top 30 most prominent GO categories are shown. (**D**) KEGG enrichment histogram. The top 30 most significantly enriched pathways are shown. (**E**) Scatter plot of KEGG] enrichment. The size of the dot is positively correlated with the number of differentially expressed genes in the pathway. Color code indicates different ranges of adjusted *p*-values.

To estimate the underlying molecular mechanism, one sample of SAA MSCs (A1) and one sample of control MSCs (C1) were randomly selected for RNA sequencing analysis. Compared to the control MSCs, 122 genes were significantly differentially expressed in the SAA MSCs. There were 49 down-regulated genes and 73 up-regulated genes (Figure 3A). To predict the cellular function of these differentially expressed genes, cluster analysis was performed to calculate the similarity scores between these genes and classify them into different clusters. As shown in Figure 3B, a clear clustering distribution was noted between the two samples. Accordingly, the SAA MSCs were quite different from the control MSCs, displaying a distinctive profile of gene expression. To elucidate the association between these differentially expressed genes in MSCs and the potential pathogenesis of SAA, Gene Ontology (GO) enrichment analysis was performed to describe the properties of these genes and their gene products in the organism (Figure 3C). Additionally, pathway functional enrichment analysis based on the Kyoto Encyclopedia of Genes and Genomes (KEGG) database was conducted to find biochemical, metabolic, or signal transduction pathways that were most potently influenced by these differentially expressed genes (Figure 3D,E).

### 2.5. MSCs from Patients with SAA Exhibiting Dysregulated TXNIP Expression

**Figure 4:**
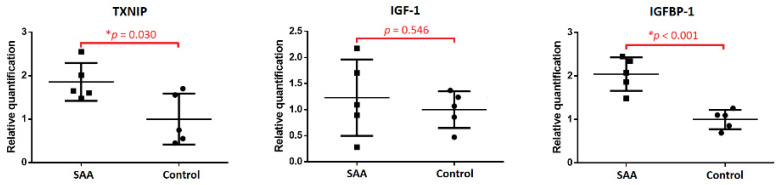
Comparison of expression of TXNIP, IGF-1, and IGFBP-1 in MSCs between patients with SAA and controls by qPCR. * Mann-Whitney U test.

A significant decrease in proliferative capacity is the hallmark of MSCs in patients with SAA. To further explore potential molecular abnormalities, we surveyed the GO terms of each differentially expressed gene in turn, especially focusing on their molecular function. Notably, the thioredoxin-interacting protein (TXNIP; ENSG00000265972) is involved in the regulation of cell population proliferation (GO:0042127), the cell cycle (GO:0007049), the positive regulation of the apoptotic process (GO:0043065), and negative regulation of cell division (GO:0000265). In our RNA sequencing study, TXNIP was significantly up-regulated in the sample of SAA MSCs (log2 fold change 4.241 and adjusted *p*-value 0.00008). We speculated that the aberrant expression of TXNIP in MSCs may be strongly associated with the disease. In consequence, qPCR was used to validate the expression of TXNIP in MSCs from five patients with SAA and five controls. Consistent with the result of RNA sequencing, the expression of TXNIP was significantly higher in SAA MSCs compared to control MSCs (*p* = 0.030; Figure 4).

For the crucial role of TXNIP in SAA MSCs, we tried to identify biological processes and pathways involved in the regulation of its expression, but there was a lack of information in the KEGG database. On the other hand, a growing body of evidence indicates a regulatory link between the insulin-like growth factor-1 (IGF-1) pathway and TXNIP [11,12]. As is known, IGF-1 is a major regulator to orchestrate cellular homeostasis, metabolism, growth, and aging. It is reasonable to associate a link with SAA. Therefore, the expression levels of IGF-1 and the insulin-like growth factor-binding protein-1 (IGFBP-1) in MSCs were determined by qPCR. While there was a wide range of IGF-1 expressions in SAA MSCs, we found that the expression levels of IGFBP-1 were significantly increased in MSCs from patients with SAA compared to those from controls (*p* < 0.001; Figure 4). 

**Figure 5:**
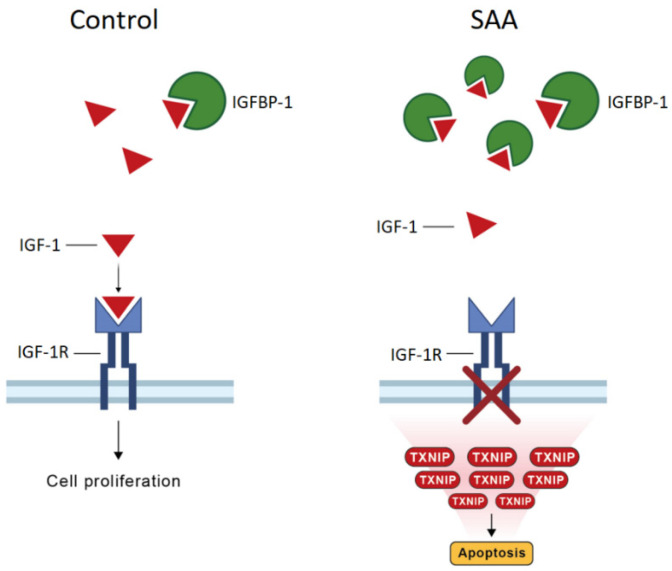
Regulation of TXNIP expression by IGF-1 and IGFBP-1. **Left** panel: the normal physiological condition. **Right** panel: the condition of SAA. Up-regulated IGFBP-1 expression leads to an increase in the binding of IGF-1 to IGFBP-1 and thus attenuates the inhibition of TXNIP expression by IGF-1. Augmented TXNIP levels may initiate deregulated cell growth and apoptosis.

Taken together, it can be assumed that a compensated increase in the expression of IGF-1 to down-regulate TXNIP in the face of SAA may be offset by the aberrantly up-regulated expression of IGFBP-1. The inhibition of TXNIP from IGF-1 may be restricted by the binding of IGF-1 to IGFBP-1. Consequently, the up-regulated TXNIP expression can induce cell death and inhibit cell proliferation, which is the hallmark of SAA MSCs. A schematic diagram of the interplay between IGFBP-1, IGF-1, and TXNIP, and the potential implications of the regulatory loop in terms of cell proliferation and apoptosis, is presented in Figure 5.

## 3. Discussion

While a significant decrease in the proliferative capacity is the hallmark of MSCs from patients with SAA, the pathophysiology and underlying molecular mechanisms are still elucidated. Our previous work demonstrated a high percentage of cells in the abnormal sub-G1 phase of the cell cycle, suggesting an increased rate of apoptosis in SAA MSCs [13]. In the present study, we found that the intensity of senescence-associated β-galactosidase activity was higher in SAA MSCs, indicating the increased senescence in these cells. Further RNA sequencing analysis identified a distinctive pattern of transcriptomes in SAA MSCs. After a survey of the differentially expressed genes, we found that the up-regulated expression of TXNIP may compromise the proliferative potential of MSCs and probably relate to the development of SAA. These results were validated by qPCR. Next, we explored the molecular mechanism involving the aberrant regulation of TXNIP in SAA MSCs. To this end, the expression levels of IGF-1 and IGFBP-1 in MSCs were measured. Despite a wide range of IGF-1 expression, the expression of IGFBP-1 was significantly increased in SAA MSCs compared to control MSCs. Accordingly, we postulated a novel regulatory link between the IGF-1 signaling pathway and TXNIP: a compensated increase in the expression of IGF-1 to down-regulate TXNIP in the face of SAA is probably offset by the anomalous expression of IGFBP-1. Consequently, an increase in TXNIP expression may induce apoptosis and inhibit proliferation, which is characteristic of SAA MSCs.

SAA is a paradigm of bone marrow failure syndromes characterized by pancytopenia in the peripheral blood and hypocellularity in the bone marrow. Despite its complex pathogenesis, the role of MSCs in the development of SAA has recently received a lot of attention. Using a long-term culture system, bone marrow stromal cells from patients with SAA were found to fail the maintenance of normal hematopoietic stem cells [14,15]. When cocultured with SAA MSCs, the proliferation of peripheral blood mononuclear cells and the colony-forming capacity of CD34+ cells significantly decreased [13,16]. As MSC-derived stromal cells are crucial in providing the specific microenvironment for hematopoiesis in the bone marrow, these data suggested a link between MSC defects and the insufficiency of hematopoietic support in SAA. As the chief defect, impaired proliferative potential is the hallmark of MSCs obtained from patients with SAA. Using population doublings as the indicator, we first reported that SAA MSCs had a lower average population doubling at each passage and a smaller cumulative population doubling from passage 4 to 6 [6]. Thereafter, the poor proliferative potential of MSCs in SAA was verified by a variety of parameters such as population doublings [16,17,18], the CCK-8 assay [18,19], growth curves [20], and colony-forming potential [21]. In our previous study, we also observed that three of five SAA MSC cultures stopped proliferating at passage five, implicating the possibility of early senescence in SAA MSCs [13]. In the present study, the higher intensity of senescence-associated β-galactosidase activity in SAA MSCs confirmed an increase in senescence in these cells. On the other hand, we first demonstrated a high percentage of cells in the abnormal sub-G1 phase of the cell cycle in SAA MSCs, suggesting an increased rate of apoptosis in SAA MSCs [13]. Our result was verified by another approach, such as an annexin V-affinity assay [19,21]. Altogether, the insufficiency of MSCs, including a decrease in their population, certainly contributes to the poor support of hematopoiesis in the bone marrow and may be an important factor in the development of SAA. 

In our previous study using microarray analysis, we found that bone marrow MSCs from children with SAA exhibited different patterns of global gene expression. Of interest, four of the five SAA MSCs samples shared a similar pattern of changes in gene expression [22]. Li et al. observed a decreased expression in the number of genes implicated in cell cycle, cell division, proliferation, chemotaxis, and hematopoietic cell lineage in MSCs from patients with SAA. Conversely, expression levels of genes related to apoptosis, adipogenesis, and immune response were elevated in SAA MSCs [21]. With the advance in experimental tools, the results of RNA sequencing analysis in the present study were consistent with the observation that SAA MSCs were quite different from the control MSCs, displaying a distinctive profile of gene expression. 

In the literature, several candidate genes were identified due to their possible association with the anomalous biological performance of SAA MSCs. Considering the contribution of MSCs to angiogenesis, down-regulated expressions of VCAM-1 and ANG-1 were proposed in MSCs from patients with SAA [23,24]. Jiang et al. found down-regulated FGF2 expression in SAA MSCs, suggesting their compromised ability for self-renewal and the impaired support of hematopoietic stem cells [20]. Considering the pivotal role of MSCs in the maintenance of hematopoietic stem cells, our previous study detected down-regulated CXCL12 expression in SAA MSCs and documented the association of its aberrant expression with the disease [22]. In the present study, we surveyed GO terms of each differentially expressed gene in the data of RNA sequencing. As TXNIP is important for the regulation of cell population proliferation, apoptosis, and cell cycle, we assumed that the up-regulated expression of TXNIP in SAA MSCs may compromise the proliferative potential of MSCs and contribute to the development of SAA. The following qPCR study validated the concept and showed the significantly increased expression of TXNIP in all of the five SAA MSCs samples.

TXNIP, which is also named thioredoxin-binding protein-2 or vitamin-D3 up-regulated protein-1, was originally discovered in HL-60 cells as a 1,25-dihydroxy vitamin-D3 responsive gene [25]. As a negative regulator to maintain the redox balance, TXNIP can directly interact with thioredoxin and inhibit its antioxidant activity. The essential physiological function of the thioredoxin system is to protect cells from oxidative damage and preserve a reduced cellular microenvironment. Thus, TXNIP is a pro-oxidant that can increase the generation of reactive oxygen species and induce oxidative stress. The overexpression of TXNIP can cause DNA damage, cell death, and autophagy-related apoptosis [26,27,28,29]. Additionally, studies have shown that the expression of TXNIP is closely related to the cell cycle process. The overexpression of TXNIP was found to arrest cell cycle progression and inhibit cell proliferation. There were a variety of cell cycle factors identified to participate in regulating TXNIP-mediated cell cycle arrest [30,31,32,33]. On the other hand, a growing number of studies have unveiled the impact of TXNIP expression on the immune system in several ways. The induction of TXNIP has been found to activate inflammatory signaling via the nucleotide-binding oligomerization domain-like receptor protein-3 (NLRP3) inflammasome, resulting in the secretion of interleukin (IL)-1β and IL-18 and, thus, facilitating inflammatory reactions [34]. TXNIPs have been demonstrated to regulate the generation, development, maturation, and functionality of various immune cells, such as natural killer cells [35], dendritic cells [36], macrophages [37], B cells [38], and T cells [39]. These findings provide evidence for the important role of TXNIP in determining the immunological makeup of the cellular microenvironment [12]. 

Considering multiple important functions of TXNIP involved in many cellular responses, the aberrant expression of TXNIP has been reported to be strongly associated with the initiation and development of various diseases in humans, such as diabetes, atherosclerosis, neurodegenerative diseases, chronic kidney disease, and many kinds of cancers [40,41]. In the present study, we found that the expression of TXNIP was significantly up-regulated in MSCs from patients with SAA. As a decrease in proliferative capacity is the hallmark of SAA MSCs, we speculated that increased TXNIP expression may play a crucial role in the compromise of proliferative potential and the promotion of aging in SAA MSCs. On the other hand, TXNIP significantly influences the immune system, and immune-mediated destruction in hematopoietic stem cells is the most widely accepted mechanism of hematopoietic failure in SAA [4]. Many studies have demonstrated the relationship between the chaotic immunomodulatory function of MSCs and the loss of immune homeostasis in the bone marrow niche in SAA [7]. In our previous study, we found aberrant cytokine profiles in the conditioned medium of MSCs from patients with SAA, with increased levels of IL-6, interferon-γ, tumor necrosis factor-α, and IL-1β [13]. Alterations in SAA MSCs on regulating T-helper 17 and regulatory T-cell differentiation were also illustrated [42]. Collectively, it is reasonable to postulate that the dysregulated expression of TXNIP in MSCs may be a pivotal factor associated with the pathogenesis of SAA.

As stated above, TXNIP is closely involved in a myriad of biological processes. Therefore, the expression of TXNIP is certainly regulated by diverse signaling pathways and biological molecules in different conditions to achieve various aims. In addition to common regulatory mechanisms such as transcriptional factors and microRNAs, TXNIP expression can also be regulated by oncogenes/tumor suppressor genes, hypoxia, endoplasmic reticulum stress signaling, and cytokines/growth factors [12]. Among these regulators, IGF-1, which is a main regulator to orchestrate cellular homeostasis, metabolism, growth, and aging, may impact TXNIP expression and be associated with MSC performance in SAA. Consequently, we measured the expression levels of IGF-1 and IGFBP-1 in SAA MSCs and control MSCs. We found a significant increase in IGFBP-1 expressions in SAA MSCs despite the wide range of IGF-1 expressions. Accordingly, we postulate a novel pathogenic mechanism of SAA: a compensated increase in the expression of IGF-1 in MSCs to down-regulate TXNIP expression in the face of SAA, which is offset by the up-regulated expression of IGFBP-1. Further studies are needed to elucidate how the regulatory link between the IGF-1 pathway and TXNIP works in this disease.

## 4. Materials and Methods

### 4.1. Patients and Controls

All patients were younger than 18 years of age. Idiopathic SAA was defined as peripheral pancytopenia with hypocellular bone marrow after excluding any other underlying diseases. The inclusion criteria of SAA were bone marrow cellularity of less than 25% and at least two of the following: absolute neutrophil count < 0.5 × 10^9^/L, platelet count < 20 × 10^9^/L, and reticulocyte count < 1% [3]. Control subjects were patients who received a bone marrow examination for diseases other than hematological diseases with pathological proof of normal bone marrow. All patients were previously untreated. The Institutional Review Board of the Chung Shan Medical University Hospital approved this study (CS2-22200), and written informed consent was obtained from the parents.

### 4.2. Isolation and Identification of MSCs 

Bone marrow MSCs were obtained and identified as in our previous reports [6,13,22,42]. Mononuclear cells were isolated from iliac crest aspirates by Ficoll-Paque density centrifugation (1.077 g/mL; Amersham Biosciences, Uppsala, Sweden). Then, cells were seeded in low-glucose Dulbecco’s modified Eagle medium (DMEM; Gibco, Gaithersburg, MD, USA) supplemented with 10% fetal bovine serum (FBS) and antibiotics/antimycotics and were incubated at 37 °Cwith 5% CO_2_ in a humidified atmosphere. The medium with the suspension of non-adhered cells was discarded after 48 h and was replenished with a fresh solution. Thereafter, the medium was replaced twice a week. Upon reaching 80–90% confluence, cells were detached with trypsin-EDTA (Gibco, Carlsbad, CA, USA) and re-plated for the subculture. Under the approval of the institutional review board, parts of the cultured MSCs were stored at −80 °C. The stored cells were thawed and cultured, and MSCs of passage 4 were used for experiments in the present study. 

The criteria of the International Society for Cellular Therapy were used for MSC identification [8]. To evaluate immunophenotypic expression, cultured MSCs were detached, washed, and resuspended in phosphate-buffered saline. After fixing and blocking, cells were immunolabeled with phycoerythrin (PE) mouse anti-human CD34 (BD Biosciences, San Jose, CA, USA), allophycocyanin (APC) mouse anti-human CD14 (BD Biosciences, San Jose, CA, USA), PE mouse anti-human CD105 (BD Biosciences, San Jose, CA, USA), or APC mouse anti-human CD44 (BD Biosciences, San Jose, CA, USA) antibodies. The nonspecific mouse IgG (BD Biosciences, San Jose, CA, USA) served as an isotype control. Data were analyzed by flow cytometry (Accuri C6; BD Biosciences, San Jose, CA, USA). To assess differentiation potential, cultured MSCs were detached and replaced in 60 mm dishes. To promote adipogenic differentiation, cells were grown in DMEM with 10% FBS, 1 μM dexamethasone, 0.5 mM 3-isobutyl-1-methylxanthine, 0.1 mM indomethacin, and 10 μg/mL insulin. For osteogenic induction, cells were grown in DMEM with 10% FBS, 10 mM β-glycerophosphate, 0.1 μM dexamethasone, and 0.2 mM ascorbic acid. After a 2-week induction, adipogenesis and osteogenesis were detected using oil red O stain (Sigma, St Louis, MO, USA) and von Kossa stain (Cedarlane, Burlington, ON, Canada), respectively.

### 4.3. Senescence-Associated β-Galactosidase Assay

To assess the senescent states of MSCs, both cytochemical and fluorescence-based methods were used to detect senescence-associated β-galactosidase activity in cells. MSCs of passage 4 were seeded into 6-well plates at a density of 1 × 10^5^ cells per well for 24 h. A senescence the β-galactosidase staining kit (Abcam, Cambridge, UK) was used to indicate the intensity of the senescence according to the manufacturer’s instructions. In brief, cultured MSCs were fixed with the supplemented fixative solution for 15 min at room temperature. Then, cells were incubated with the freshly prepared staining solution at 37 °C overnight and finally observed under an inverted light microscope. The Fluorescence Senescence Assay Kit (Abcam, Cambridge, UK) was used for the fluorescence detection of senescence-associated β-galactosidase activity in MSCs according to the manufacturer’s protocol. Briefly, cultured MSCs were plated on coverslips overnight. Then, cells were incubated with the senescence dye solution at 37 °C for 2 h. After being washed with the buffer solution, MSCs were fixed with 4% paraformaldehyde and observed under a fluorescence microscope. To quantify the fluorescence levels, cells were collected by trypsinization and analyzed by flow cytometry (Accuri C6; BD Biosciences, San Jose, CA, USA).

### 4.4. RNA Sequencing and Bioinformatics Analysis

Total RNA was extracted using the PreAnalytiX RNA extraction kit (Qiagen, Hilden, Germany) from the MSCs of passage 4. Following extraction, the concentrations of RNA samples were measured spectrophotometrically at an optical density of 260/280 (NanoDrop Technologies, Wilmington, DE, USA). For transcriptome sequencing, 1 μg of total RNA was used for library preparation according to the manufacturer’s instructions. Then, the prepared libraries with different indexes were multiplexed and loaded onto the Illumina NovaSeq Platform (Illumina, San Diego, CA, USA) for sequencing using a 2 × 150 paired-end configuration).

To obtain clean high-quality, pass-filtering data, they were processed by Cutadapt (version 1.9.1) to remove adapters, 5’ or 3’ ends containing bases of quality values below 20, and reads that were less than 75 bp long after trimming. After quality control, clean data were aligned to an annotated reference genome via the HISAT2 program (version 2.2.1), and the HTSeq (version 0.6.1) was used for quantifying gene expression levels. Differential expression analysis was performed using the DESeq2 package (version 1.6.3), with genes considered significantly differentially expressed if the log2 fold change was greater than 2 and the adjusted *p*-value was less than 0.05.

Furthermore, hierarchical cluster analysis was performed using the gplots package in R (version 2.3.2) to calculate and classify data according to similarity. To provide information on how the differentially expressed genes were related to certain biological functions, the GOseq (version 1.34.1) was used to conduct GO enrichment analysis, and the TopGO package (version 2.18.0) was applied to test GO terms while accounting for the topology of the GO graph. Additionally, pathway functional enrichment analysis based on KEGG pathway units was used to determine the most biological pathways that the differentially expressed genes were involved in.

### 4.5. Quantitative Real-Time PCR (qPCR)

For qPCR, total RNA was extracted from MSCs of passage 4 as previously described in procedures. cDNA was then synthesized using the cDNA Reverse Transcription Kit (Applied Biosystems, Foster City, CA, USA). The sequences of primers were as follows: TXNIP, forward 5′-AGGGGTGTTTGTTGGATGGG-3′ and reverse 5′-TTACGCCAGGAGGCCATTTT-3′; IGF1, forward 5′-CTCTTCAGTTCGTGTGTGGAGAC-3′ and reverse 5′-CAGCCTCCTTAGATCACAGCTC-3′; IGFBP1, forward 5′-TGGATGTAGACCCAGAGAGCA-3′ and reverse 5′-GGCGCTCACACTTGAAAAAC-3′. The expression of GAPDH (forward 5′-ACCCACTCCTCCACCTTTGACG-3′ and reverse 5′-TCTCTTCCTCTTGTGCTCTTG-3′) was used as the internal control. According to the manufacturer’s instructions, qPCR was performed using cDNA samples with SYBR Green PCR Master Mix on the ABI 7300 Real-time PCR system (Applied Biosystems, Foster City, CA, USA). Fold differences were calculated by the 2^ΔΔCT^ method. All samples were performed in triplicate. Finally, data analysis was performed using SPSS 16.0 for Windows, and the Mann–Whitney U test was used to compare groups. A value of *p* < 0.05 was considered statistically significant.

## Figures and Tables

**Table 1:** Clinical characteristics of the participants.

Patient No.	Gender	Age at Diagnosis (Years)	Diagnosis
Five patients with SAA			
A1	Male	17.1	Idiopathic SAA
A2	Female	14.7	Idiopathic SAA
A3	Male	9.3	Idiopathic SAA
A4	Female	12.2	Idiopathic SAA
A5	Male	10.3	Idiopathic SAA
Five controls			
C1	Male	17.8	Kikuchi disease
C2	Male	16.3	Rhabdomyosarcoma, stage I
C3	Female	5.9	Ewing’s sarcoma, stage I
C4	Female	15.0	Rhabdomyosarcoma, stage I
C5	Male	2.8	Hepatoblastoma, stage I

SAA: severe aplastic anemia.

## Data Availability

The data presented in this study are available on request from the corresponding author.
